# Non-ST-Segment Myocardial Infarction Resulting From Thrombotic Saphenous Vein Graft Aneurysm

**DOI:** 10.7759/cureus.14227

**Published:** 2021-03-31

**Authors:** Nicholas L Biondi, Ahmed Nour, Bryan J Fiema, Ravi S Akula

**Affiliations:** 1 Adult Cardiology, Arnot Ogden Medical Center, Elmira, USA; 2 Graduate Medical Education, Lake Erie College of Osteopathic Medicine, Elmira, USA; 3 Internal Medicine, Arnot Ogden Medical Center, Elmira, USA

**Keywords:** adult cardiology, great saphenous vein, general internal medicine, non-st-segment elevation myocardial infarction (nstemi), coronary artery bypass grafting (cabg), acute coronary syndrome

## Abstract

Acute coronary syndromes from coronary emboli are rare, but well described in the literature. Saphenous vein grafts (SVG), used in coronary artery bypass grafting surgery, are vessels prone to atherosclerotic occlusion and aneurysmal dilation. Descriptive cases of embolization of these atherosclerotic lesions are lacking. Aneurysmal dilation of SVG conduits provides an area of stagnated flow that can harbor thrombi with embolic potential. This case describes a patient with non-ST-segment myocardial infarction possibly resulting from SVG aneurysm thrombus embolism.

## Introduction

Coronary artery bypass grafting (CABG) is a procedure done more than 200,000 times annually [1] to treat severe coronary artery disease (CAD) and improve survival [2]. Although this rate is declining overall [1], it remains a reliable tool in the arsenal of cardiologists for the treatment of severe CAD [2]. CABG therapy has demonstrated a survival advantage and anginal symptom reduction when compared to medical therapy for patients with left main or three-vessel CAD [2]. Initial CABG surgeries utilized left internal mammary arteries (LIMA) and right internal mammary arteries as the main bypass conduits [3]. Dr. Rene Favaloro, of the Cleveland Clinic, experimented with the use of saphenous vein grafts (SVGs) as bypass conduits performing the first CABG surgery with an SVG in 1967 [3].

SVG may undergo aneurysmal dilation which can impact surrounding cardiac structures and increase morbidity and mortality [4]. The first case of SVG aneurysm (SVGA) was reported in 1975 [4]. Aneurysmal dilation and SVG failure have largely been attributed to atherosclerotic cardiovascular disease, but no definitive cause has been confirmed [2-5]. Other theories hypothesize that the physical attachment of a vein, denuded of its vasa vasorum, to the high-pressure arterial system at its largest, and consequently the weakest, diameter are underlying mechanisms leading to aneurysmal dilation [4]. SVGA has been linked to a wide array of clinical phenotypes: chest pain/angina, dyspnea, myocardial infarction (MI), heart failure, syncope, shock, hemoptysis, arrhythmia, and sepsis [4]. Although aneurysm rupture is a feared complication owing to poor prognosis, it is scarcely reported in the literature [4]. SVGA may also harbor thrombi with the potential for flow occlusion or distal embolization [4]. Predictably, event rates increase with increasing aneurysm size. The driving factor for the increased event rate pertains to mechanical complications as opposed to infarction, rupture, or death [4]. Event rates for aneurysms less than 20 mm in diameter approach 33% while those for aneurysms over 100 mm surpass 70%, implicating the need for standardized surveillance and management plans [4].

## Case presentation

Our patient was a 78-year-old male presenting for evaluation of acute-onset, anginal chest pain of six-hour duration. The pain was typical of classic anginal chest pain with radiation to the left neck. The patient had an additional complaint of a diffusely edematous, ecchymotic left lower extremity (LLE); however, he was recently post-operative from a left total knee arthroplasty (TKA) four days prior.

The patient was maintained on warfarin for a previously identified SVGA with associated thrombus. His warfarin was discontinued three days before the surgery, and an enoxaparin bridge was started instead. The warfarin was restarted on postoperative day one without a bridging strategy at an initial international normalized ratio of 1.1.

The patient was previously well with a past medical history notable for three-vessel CABG in 2017 [LIMA to left anterior descending (LAD), SVG to right coronary artery (RCA), and SVG to first diagonal (D1)], SVG-D1 aneurysm with thrombus, thrombocytopenia, essential hypertension, mixed dyslipidemia, New York Heart Association [6] class III/American Heart Association class C heart failure with reduced ejection fraction, IgA-kappa monoclonal gammopathy of undetermined significance, and osteoarthritis. In addition to the CABG, the patient had undergone bilateral TKA, Roux-en-Y gastric bypass, and cervical fusion of C4-C6.

The patient presented afebrile with stable vital signs: a pulse of 73 beats per minute and blood pressure of 123/57 mmHg, saturating 94% on room air at 18 breaths per minute. On examination, the patient had a prominent S4 gallop and a markedly ecchymotic LLE with 3+ pitting edema to the mid-thigh without jugular venous distension. Initial laboratory findings were remarkable for anemia with a hemoglobin of 6.9 g/dL (baseline 13.5 g/dL), thrombocytopenia to 127 × 103/µL, and an initial high sensitivity troponin of 27 pg/mL.

**Figure 1:**
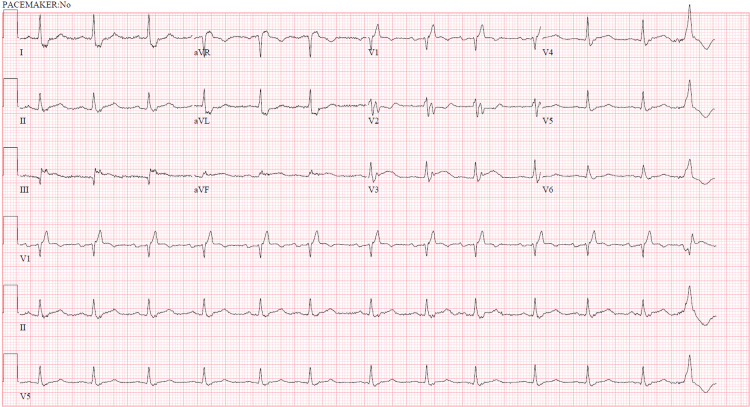
Presenting electrocardiogram revealing normal sinus rhythm and non-specific ST-segment changes secondary to complete right bundle branch block. There were no significant interval changes compared to previous electrocardiographic tracings.

Following evaluation by emergency department personnel, the patient was placed for admission for suspected non-ST-segment myocardial infarction (NSTEMI) with presenting electrocardiogram shown in Figure 1.

**Figure 2:**
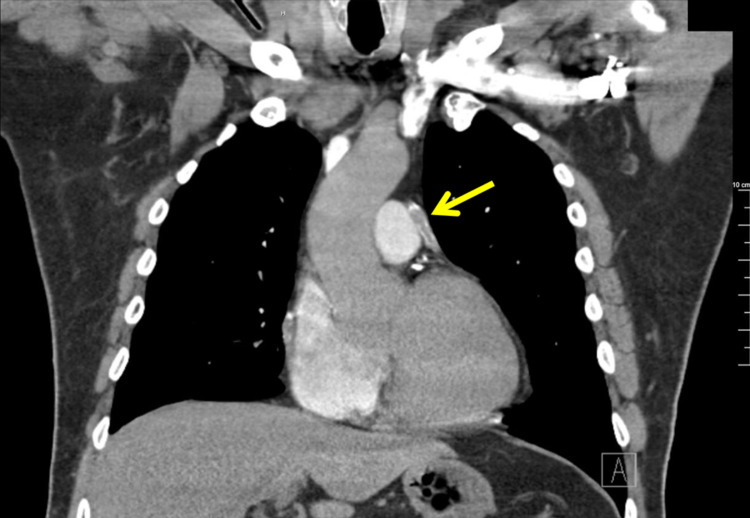
Computed tomography angiogram of the chest demonstrating calcific disease in the SVG to the first diagonal branch of the LAD coronary artery. SVG, saphenous vein graft; LAD, left anterior descending

The high-sensitivity troponin increased steadily peaking at 9,632 pg/mL before downtrending. Acute coronary syndrome (ACS) protocols were enacted and the patient was started on a heparin drip. The patient was transfused with packed red blood cells for his anemia. Computed tomography (CT) angiogram of the chest identified known SVG to D1 disease (Figure 2) without the presence of pulmonary embolus. The chest pain improved with the therapies and the patient consented to cardiac catheterization.

**Figure 3:**
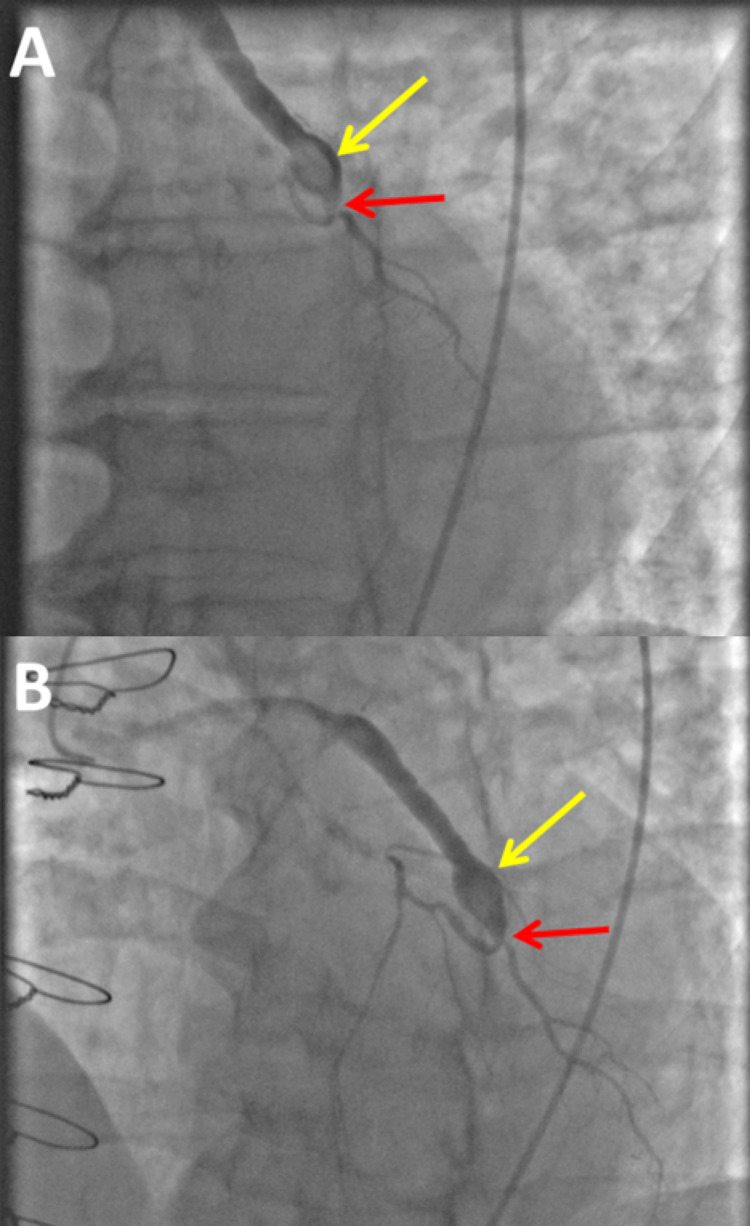
Invasive coronary angiographic comparison of the SVG to the first diagonal branch of the LAD coronary artery (D1) between March 2016 and October 2020. Aneurysmal dilation of the SVG is largely unchanged with minimal interval worsening of the D1 anastomosis site. The yellow arrows highlight the SVC aneurysm with organized thrombus, while the red arrows highlight the distal anastomotic site of the SVG to D1. (A) March 2016 coronary angiogram with a selective injection of the SVG to D1 graft. (B) October 2020 coronary angiogram with a selective injection of the SVG to D1 graft. SVG, saphenous vein graft; LAD, left anterior descending; SVC, superior vena cava

Cardiac catheterization revealed severe native vessel CAD involving the proximal LAD, proximal RCA, and ostial D1. The bypass grafts were patent, and the distal SVG-D1 anastomosis was aneurysmal with an organized thrombus without significant change compared to March 2016 (Figure 3). Post-catheterization diagnosis was suspected distal embolization of a thrombus fragment resulting in branch vessel occlusion resulting from warfarin. The patient tolerated the procedure without incident. The remainder of his hospital course was uneventful. He was discharged home in a stable condition on hospital day four on guideline-directed medical therapy for his cardiac ailments.

## Discussion

A relatively rare yet clinically significant complication of CABG is the development of aneurysmal dilatation of the harvested SVG, which is most commonly grafted to the RCA [4]. When grafted to the LAD coronary artery, SVGA form in approximately 25% of the cases [4]. SVGA is typically diagnosed 10 years or more after bypass surgery [4]. The development of SVGA is further characterized into early SVGA, 16 days to several months post-CABG, and late SVGA, five or more years following the surgery [4]. Early SVGA is likely a consequence of infection, intrinsic vessel wall weakness, or harvesting process errors, while late aneurysmal dilatation is thought to result from atherosclerotic degeneration compromising vessel wall architecture [2-5,7]. Approximately 10% of SVGA detected are classified as early, with 4% occurring within one year and the remaining 6% occurring between one and five years. Aneurysms detected between five and ten years post-surgery account for an additional 20% of the cases [4]. The overall incidence of SVGA development is likely underestimated due to their asymptomatic nature [4].

SVGA can result in MI, rupture, death, and mechanical complications [4]. No consensus exists regarding the approach to diagnosing or managing SVGA [4]. Current best practices rely on multi-slice CT [8], magnetic resonance imaging, and cardiac catheterization for accurate identification of SVGA [4]. Cardiac catheterization is prone to false-negative results if a concomitant aneurysmal thrombus is present [4]. Vein grafts are also prone to occlusion [2,3]. Approximately 10-25% occlude within one year, and others experience an annual occlusion rate of 1-2% within one to five years and 4-5% within six to ten years [2]. Overall, only 50-60% of SVG are patent at 10 years [2].

Treatment guidelines are similarly ambiguous for SVGA [4]. No definitive data exist delineating the benefit between surgical intervention, resection or ligation, and conservative, medical approaches for asymptomatic patients. In the cases of significant myocardial compromise and infarction, further bypass grafting may be indicated. For symptomatic patients, those with SVGA dilation greater than 1 cm, and those with compromised SVG graft flow, surgical revascularization carries a significant benefit [5].

Our case presents a thrombosed SVGA causing NSTEMI through suspected distal embolization. There are many case reports of MI resulting from mechanical compression of the myocardium [9,10], but none report distal thrombus embolization from SVGA. Coronary embolization (CE) is an established cause of 4-7% of MI [11,12], and was first described by Virchow in 1856 [11]. These embolized thrombi and plaque debris expand infarct territory [11] and worsen outcomes. Embolic phenomena resulting in MI have been associated with valvular prostheses, rheumatic valvular disease, chronic atrial fibrillation, dilated cardiomyopathy, intracardiac shunts, intracardiac tumors, infective endocarditis, and hypercoagulable states [12,13] SVGA with concomitant thromboses have a theoretical potential for embolization resulting in downstream vessel occlusion and infarction. Interventional strategies are not well established for managing this subtype of CE [13]. Strategies including percutaneous coronary intervention, balloon angioplasty, thrombectomy, and thrombolysis are some proposed modalities for treating MI caused by CE [12-14].

Focusing on the source of our embolization, little data is available on optimal treatment strategies. The use of oral anticoagulant medications for the treatment of SVG thrombosis has been proposed. Warfarin use has previously been studied for effect on long-term graft patency, but not for thrombus treatment [15]. Extrapolation of deep venous thrombosis treatment has been applied to patients with SVG thromboses, and the use of direct oral anticoagulant mediations has been proposed as a viable option for SVG thrombus resolution [16]. More data are needed before definitive conclusions regarding management can be made.

## Conclusions

This case proposes that SVGA can represent an unrecognized source of CE and subsequent infarction in post bypass patients. Early identification and treatment of thromboses with oral anticoagulant medications may help decrease embolic potential. Management strategies devised specifically for these rare instances are lacking. As such, the application of formalized ACS guidelines should be used.
